# A narrative review of veterinary pharmacovigilance situations and prospects in East African countries

**DOI:** 10.3389/fvets.2024.1430587

**Published:** 2024-11-01

**Authors:** Yesuneh Tefera Mekasha, Sete Nigussie, Abibo Wondie Mekonen, Kassahun Berrie, Asnakew Mulaw, Melaku Getahun Feleke

**Affiliations:** ^1^Pharmaceutical Science, Pharmaceutical Quality Assurance, and Regulatory Affairs, University of Gondar, Gondar, Ethiopia; ^2^Department of Veterinary Pharmacy, College of Veterinary Medicine and Animal Sciences, University of Gondar, Gondar, Ethiopia; ^3^Department of Veterinary Pharmacy, Drug Supply Chain Management, University of Gondar, Gondar, Ethiopia; ^4^Veterinary Epidemiology and Public Health, College of Veterinary Medicine and Animal Sciences, University of Gondar, Gondar, Ethiopia; ^5^Veterinary Pathobiology, College of Veterinary Medicine and Animal Sciences, University of Gondar, Gondar, Ethiopia; ^6^Department of Veterinary Pharmacy, Pharmaceutical Analysis, and Quality Assurance, University of Gondar, Gondar, Ethiopia

**Keywords:** review, veterinary drug, pharmacovigilance, drug safety, East Africa

## Abstract

**Background:**

Veterinary pharmaceuticals must be safe and effective for treating and preventing diseases in animal sectors. Strict adherence to regulations at every stage of production, storage, and administration is necessary as the global sector grows in order to lower the possibility of adverse veterinary drug events. Strong pharmacovigilance regulatory systems are critical in monitoring and managing safety concerns related with veterinary pharmaceuticals.

**Objective:**

This review aimed to investigate the implementation of veterinary pharmacovigilance, collaborative initiatives, and reporting practices within the veterinary sector across East African countries.

**Methods:**

A thorough search was performed using online platforms such as Google Scholar, PubMed, the Web of Sciences, and regulatory Web sites. The search strategies relied heavily on selecting relevant published findings related to veterinary pharmacovigilance status, veterinary adverse drug event reporting practice, and collaborative efforts in veterinary pharmaceutical sectors within the East African landscape. This article search approach confirmed the inclusion state of veterinary pharmacovigilance and associated collaborative initiatives in the region.

**Results:**

In comparison to more developed regions, the review indicated that the veterinary pharmacovigilance system in East African countries was still in its early state. A strong legislative foundation and a large commitment from the veterinary profession are needed to establish a nationwide veterinary pharmacovigilance system. The review reveals a significant lack of consistency in the infrastructure of veterinary pharmacovigilance among the countries of East Africa. Tanzania, Kenya, and Ethiopia have some institutional processes for veterinary pharmaceutical safety, but they lack thorough documentation, which suggests that this systems still require improvement. The analysis emphasizes how inadequate the reporting systems are for adverse veterinary medication events in the majority of East African nations. Given the challenges East Africa faces, customized strategies are required to guarantee the safety and oversight of veterinary medications and improve veterinary pharmacovigilance. For systems to be more effective, veterinary pharmaceutical legal frameworks must be strengthened and stakeholder collaboration must be improved. Stakeholders include legislators, law enforcement, customs officials, regulatory organizations, scientists, pharmaceutical businesses, veterinarians, farmers, and the general public.

**Conclusion:**

A multidisciplinary strategy is needed to solve current gaps in veterinary pharmacovigilance and improve overall veterinary drug safety in East Africa. It is feasible to create more reliable methods for tracking and guaranteeing the safety of veterinary medications by combining the efforts of numerous stakeholders, including as legislators, regulatory agencies, veterinary practitioners, and the general public.

## 1 Introduction

Veterinary medicines have been used in the treatment, control, and prevention of animal diseases (1). However, the safety and effectiveness of these medications are determined by their quality, which is maintained throughout the production process, shelf life, and administration of the products (2). Despite the fact that the global veterinary product manufacturing industry is growing, there is also evidence that continues to mount that adverse reactions to medicines are a common problem (3). Adverse effects in animals frequently occur, which is often associated with off-label use of medication, incorrect dosage or administration, and poor-quality medications (4). Currently, treatment failure, adverse drug reactions, drug resistance, drug residue, and deaths are the most frequent events and are primarily associated with the safety and utilization practices of veterinary medications (5). The safety issues of medication are becoming a challenge for global health systems and pharmaceutical companies (6). The extent of the challenge is influenced by a number of factors, including regulatory body capabilities, stakeholder cooperation, national resource availability, and level of professional skills or experience (7).

The developed nations are overcoming the challenge by establishing systems (8) to ensure the safety of medicines. However, this system is not implemented in the case of developing countries (6). In Africa, in the past three decades, there has been an increase in both the manufacturing and utilization of veterinary pharmaceuticals; however, systems to monitor and ensure their safety are still impractical and are a major problem (6). In developing nations, particularly in African countries, pharmacovigilance is being implemented in health systems with poor infrastructure, unreliable supply and quality of medicines, lack of adequately trained health staff, poor awareness and interest of national stakeholders in drug safety, poor regulatory capacity, and limited funding for health-related concerns by national authorities (9). Due to insufficient veterinary laboratory services, performing confirmatory diagnosis of disease-causing agents is very challenging for East African countries (7). This leads to incorrect diagnoses and inappropriate use of veterinary drugs, resulting in undesirable therapeutic effects such as adverse drug reactions, harmful interactions, toxicity, and drug resistance. Therefore, spontaneous reporting of suspected adverse drug reactions is crucial for pharmacovigilance. It is an essential tool for physicians to navigate these challenges and determine the best and safest medication(s) for individual animal patients, following established safety registration formats (3). In spite of this, it is thought that adverse drug reactions related to veterinary medications were significantly underreported (10). In the event of safety issues, low reporting rates can delay the response of regulators and marketing authorization holders (MAHs) in implementing mitigation actions (10). Unintended drug effects can result from a number of factors, including issues related to the drug itself—such as overdosing, under dosing, polypharmacy, or counterfeit and substandard medications—as well as patient-related factors like age and species differences, and clinical issues such as improper administration and misdiagnosis. The major problem is that these factors are often not properly registered and reported formally (11).

Evidence indicates that several adverse effect records were documented for many pharmaceutical classes of medications after being administered to animals. However, such drug responses remain largely unrecorded in developing nations (12). In veterinary medicine, antibiotics, antiparasitics, and non-steroidal anti-inflammatory drugs are the most frequent medications that are linked to adverse effects and/or poisoning (13). Existing studies indicated that frequent adverse effects of medication mainly impact companion animals, rather than farm animals. For farm animals, fewer cases were reported (13). For instance, benzimidazoles are popular anthelmintics that are used extensively in many animal species, but if not used safely, they are poisonous to the bone marrow and the mucosa of the gut. This toxicity occurs due to their inhibition of mitosis, even though differences between worm and mammalian tubulin exist (14). Nephrotoxic and hair loss are adverse effects described following thiabendazole administration in dogs (14). Additionally, levamisole is considered a drug of anthelmintics with a narrow therapeutic index and has many possible adverse and toxic effects, such as subsequent decreased convulsion threshold, paralysis of respiratory muscles, and asphyxia in many animal species (15). Several adverse reactions are associated with off-label use of metronidazole, such as anorexia, nausea, vomiting, and diarrhea; neurological symptoms, particularly vestibular and cerebellar dysfunction; blood count alterations; and toxic effects on the liver (16). Certain antimicrobials such as tetracyclines and macrolides have a considerable risk of causing tissue damage in cattle when administered intramuscularly (17).

A particular problem associated with a solution for infusion was a known adverse effect in cows with milk fever: here, infusion with calcium and/or magnesium, which was administered to treat hypocalcemia and hypomagnesia, can lead to cardiac arrest if the dosage was too high (18). The ototoxic antimicrobial substance gentamicin is frequently reported in relation to deafness in dogs (18, 19). Hypersensitivity reactions and anaphylaxis have been reported with vaccines in both cats and dogs. In addition, hypersensitivity is the main problem of dogs following the administration of antimicrobials, such as sulphonamides and potentiated sulphonamides (20).

A study indicated that in one instance, over 35,000 sheep were treated with the endectocides ivermectin, and over 600 died. It was found that incorrect administration was responsible, and as a result, severe damage to the throat occurred, which led to the deaths observed (18). Usually, young cattle in particular have collapsed after treatment with some corticosteroid and analgesic preparations. The reasons for these effects were not fully evident (21). A study in Sweden showed that adverse reactions to vaccines in dogs were relatively common, while in horses, the majority of adverse reactions were to antimicrobial drugs; three horses died following treatment with trimethoprim sulfadiazine products, and seven died after administration of benzylpenicillin (18).

A survey in Sub-Saharan Africa found 38% of adverse reactions in animals, 24% in efficacy in animals, and 38% in humans. The main animal species affected were dogs (44%), poultry (24%), and pigs (22%). Antiparasitics (61%) were the most reported class of drugs associated with adverse reactions in animals, followed by antibiotics (24%). Adverse effects occurred in 52% of cases following veterinary drug use in accordance with the manufacturer’s instructions and in 35% after off-label use (3). Establishing a robust veterinary pharmacovigilance system is essential for safeguarding animal health and ensuring the safety and efficacy of veterinary medicines. A pharmacovigilance system helps in identifying adverse drug reactions (ADRs) early, allowing for timely interventions and minimizing harm to animals (22). This is a key component of effective drug regulation systems, clinical practice, health programs, and other stakeholders (3). A veterinary pharmacovigilance (PV) system is a tool for any veterinary drug regulatory agency that has the responsibility of having a well-established system to monitor adverse drug reactions (ADRs) during the drug development phase and later during the life of a marketed drug (22). In East Africa, the documentation and monitoring of veterinary adverse drug events (ADEs) are often insufficient, which poses significant challenges. Therefore the objective of this review was to provide an overview of the status of veterinary pharmacovigilance in the selected East Africa.

### 1.1 Definitions of terms

**Veterinary pharmacovigilance:** Veterinary pharmacovigilance is a process by which information is collected and analyzed to detect and prevent unexpected or unwanted adverse effects following the use of veterinary medicinal products (23).

**Active surveillance system** refers to the collection of case safety information as a continuous pre-organized process (24).

**Post-marketing surveillance** refers to applied adverse drug reactions (ADRs) from the post-endorsement stage and all through a medication’s market life (25).

**Adverse event in animals** is defined as any observation that follows the use of veterinary medications (including off-label and on-label usage), regardless of whether it is thought to be connected to the product (26).

**A serious adverse event** is any incident that causes a congenital deformity or anomaly, resulting in a life-threatening injury or incapacity, or results in death (27).

**An unexpected adverse event** is one whose nature, severity, or result does not match the authorized labeling or approved documentation that outlines the predicted adverse effects of veterinary medications (27).

## 2 Methodology for data search and extraction

### 2.1 Databases and search strategy

The review was performed between January and April 30, 2024, with an emphasis on published literatures and gray literature, mostly from East Africa. The Web of Science, PubMed, Google Scholar, and regulatory websites were among the databases evaluated for this review. Keywords and search phrases were utilized to gain pertinent data. “East Africa,” “Veterinary Pharmacovigilance,” “Veterinary Drug Surveillance Program,” “Veterinary Drug Safety OR Adverse drug event,” “Adverse Drug Reactions Reporting Systems,” and “Veterinary Post-Marketing Surveillance” were among the terms and phrases that were employed. Boolean operators were employed both singly and in conjunction with these terms to improve and adjust the search results’ accuracy. The search approach was designed to find a wide variety of research and reports relevant to the goals of the study.

### 2.2 Inclusion and exclusion criteria

The study included in this review encompassed only those published and gray literature sources that provided information on veterinary pharmacovigilance systems, adverse drug reactions, regulatory framework, reporting systems, and the monitoring of veterinary drug-related side effects conducted in East Africa. For making future policy recommendations, evidence regarding adverse drug reactions, vigilance systems, and any drug-related evidence was also included. Published papers that did not specifically contain information on the veterinary pharmacovigilance environment, particularly those focused on human environments, were excluded from the review. The search strategies and data extraction processes were conducted independently by four individuals: YTM, SN, AW, KB, AM, and MGF for data quality and methodological validity. Any conflict was resolved by discussing with YTM.

## 3 Literature search results

### 3.1 Trends in veterinary pharmacovigilance and reporting practices: lessons from East Africa

In this review, East African veterinary pharmacovigilance practices were examined to identify gaps and provide future recommendations for enhancement. Veterinary pharmacovigilance involves the systematic collection, analysis, and prevention of adverse effects associated with the use of veterinary medicinal products (23). An adverse event refers to any unintended and unfavorable observation in animals or humans following exposure to veterinary products. This includes a lack of expected efficacy and any negative events following off-label usage (26).

National medicine regulatory authorities are responsible for ensuring the safety and quality of medicines, including veterinary products (2, 28, 29). These authorities are expected to prevent the circulation of falsified and substandard medicines. However, in Africa, no single national body has the complete capacity to manage all regulatory responsibilities (30). The organizational setup and functionality of these bodies vary significantly across the continent. Moreover, animal health receives far less attention compared to human health, with regulatory frameworks often not specifically tailored to veterinary products (31, 32).

According to the World Organisation for Animal Health (33), while several African nations have legislation concerning pharmaceutical products, these laws differ greatly and are often inadequately implemented (34). This lack of consistent regulation affects pharmacovigilance, leading to issues like duplication of efforts and gaps in ensuring public health and safety.

Based on the currently available reports on the OIE website for 20 countries of Sub-Saharan Africa, an excessive number of veterinary medications are supplied without a prescription due to the public supply chains being uncontrolled from import to retail due to repeated failures in veterinary administration and regulation (35). As a consequence, veterinary drugs can be found anywhere, anyhow (36). The improper distribution of veterinary drugs and off-label product usage by non-veterinarians account for a sizable number of adverse events that have been reported to the Veterinary Pharmacovigilance Center in South Africa (37).

In East Africa, the registration, distribution, and usage of veterinary medicines are often governed by pharmacy boards or equivalent bodies. However, these organizations frequently lack the necessary funding and manpower to fulfill their mandates effectively. This results in the uncontrolled importation and distribution of veterinary medicines, contributing to the widespread availability of these drugs without proper prescriptions and oversight (38).

### 3.2 Pharmacovigilance reporting practices

Literature indicated that veterinary medications were supplied without prescriptions due to weak regulation of the veterinary pharmaceutical services (39). The improper distribution, coupled with off-label use by non-veterinarians, results in numerous adverse events. For example, in South Africa, a significant number of adverse events reported to the Veterinary Pharmacovigilance Center stem from such practices.

In most of the nations in Eastern Europe, the registration, distribution, and usage of veterinary medications are governed by pharmacy boards (or equivalents); however, it has been reported that these organizations lack the funding and manpower necessary to carry out their mandate (40), leading to unrestricted importation and distribution of veterinary medicines has been found in the areas (40). In East Africa, the legal frameworks range from Acts of Parliament as found in Kenya, Uganda, Tanzania, and Malawi to Ministerial decrees or proclamations being used in Ethiopia (40). Tanzania and Kenya have relatively up-to-date laws governing the provision of veterinary services, and they have set up regulatory bodies that specify the precise responsibilities of veterinary boards, veterinary surgeons, veterinary paraprofessionals, animal owners, and private veterinary practices. Ethiopia is in the initial stages of setting up a proclamation to establish a veterinary board to regulate the training and registration of veterinarians and the delivery of veterinary services (40). The status of veterinary pharmacovigilance guidelines from selected East African countries was elaborated:

#### 3.2.1 Report of adverse events: lesson from Ethiopia

The Ethiopian Agricultural Authority (EAA) has been proactive in establishing and enforcing veterinary pharmacovigilance, operating under the legal mandate of Proclamation 728/2011, Article 7. This mandate empowers the EAA to conduct post-marketing surveillance to evaluate the benefits and harms of registered veterinary drugs. The primary focus of veterinary pharmacovigilance is to ensure the safety and efficacy of drugs in animals and the safety of people exposed to these products (12). However, despite these efforts, the implementation of veterinary pharmacovigilance in Ethiopia remains in its early stages. Several adverse drug events have been reported, highlighting the challenges and importance of this system:

*Diazinone-Related Deaths:* There have been instances of 30 deaths attributed to incorrect dosage preparation of diazinone, underscoring the need for precise dosing instructions and better training for those administering the drugs (12).

*Oxytetracycline 10%:* Adverse events have also been linked to the administration of oxytetracycline 10%, pointing to potential issues with this particular formulation or its usage in practice. Drug Interactions: There have been reports of deaths resulting from the simultaneous administration of two drugs, indicating a critical need for better guidelines and awareness regarding drug interactions.

*Dose of ivermectin (0.2 mg/kg or 1 mL/50 kg):* Ivermectin, when administered to calves, showed a significant impact on Anopheles arabiensis mosquitoes. During the follow-up period, lasting until day 21, ivermectin treatment resulted in substantially higher mosquito mortality rates than control groups (41). On the first day following treatment, the average 24-h mortality rate of An. arabiensis was 81.6%. Moreover, 100% of the An. arabiensis mosquitoes that fed on ivermectin-treated calves on the first day after treatment died within 4 days. Additionally, the egg production rate of An. arabiensis fed on ivermectin-treated calves was significantly lower than that of the control group. This indicates that ivermectin not only increases mortality in An. arabiensis but also reduces their reproductive capacity, potentially contributing to the control of mosquito populations and the diseases they transmit. To improve the situation, it is essential to enhancing the regulatory framework and putting strong pharmacovigilance practices in place are necessary to improve the situation. These steps include: (a) training and education; (b) creating a user‑friendly, effective system for reporting adverse drug reactions and making sure that all relevant parties understand when and how to report such incidents; (c) creating clear guidelines on drug interactions and safe dosage practices to prevent avoidable adverse events; and (d) educating the public and animal owners about the potential risks associated with veterinary medications and the significance of following prescribed dosages and guidelines. By addressing these issues, the EAA may improve Ethiopia’s veterinary pharmacovigilance program, ultimately safeguarding the security and welfare of both people and animals.

*Training and Education:* Providing comprehensive training for veterinarians and animal health workers on proper drug administration and the importance of reporting adverse events.

*Reporting Systems:* Establishing an efficient and accessible system for reporting adverse drug reactions and ensuring that all stakeholders are aware of how and why to report such incidents.

*Guidelines and Regulations:* Developing clear guidelines on drug interactions and safe dosage practices to prevent avoidable adverse events.

*Public Awareness:* Raising awareness among the general public and animal owners about the potential risks of veterinary drugs and the importance of adhering to prescribed dosages and guidelines.

By tackling these areas, the EAA can strengthen the implementation of veterinary pharmacovigilance in Ethiopia, ultimately ensuring the safety and wellbeing of both animals and people (12).

#### 3.2.2 Report of adverse events: lesson from Kenya

The Kenya Veterinary Board (KVB) is responsible for regulating veterinary practice and licensing veterinary professionals (39). The board plays a critical role in setting standards for veterinary services and ensuring that practitioners adhere to these standards.

Kenya is grappling with a significant issue related to counterfeit and adulterated drugs, accompanied by notable quality control challenges. Industry members estimate that approximately 30% of all drugs in Kenya are substandard (42). A recent study by the Kenya Agricultural Research Institute (KARI) highlighted that while injectable products were typically of good quality, many anthelmintics and pour-on acaricides were found to be substandard. Due to these issues, livestock owners in some regions experience considerable problems with ineffective drugs, particularly inexpensive generic products. This has led them to increasingly seek new products as well as education and advice from reputable companies. When adverse reactions or product failures occur, these are typically reported directly to the supplier by the distributor. However, most suppliers attribute these events to misuse or adulteration by the retailer or end-user rather than defects in the products themselves (42). The need for improved regulatory measures, quality control, and education to ensure the efficacy and safety of veterinary drugs is warranted.

#### 3.2.3 Report of adverse drug event: lesson from Sudan

Sudan has no independent veterinary product regulatory body rather single agency called The National Medicines and Poisons Board (NMPB)^1^ under the Ministry of Health has the mandate to regulate food, human drugs, veterinary drugs, and medical devices and to ensure adequate and effective standards (43). Even the practice of the agency was less in appointing inspectors to monitor the importation, distribution, or sales of veterinary drugs.

It seems that oxytetracycline, when administered at a dose rate of 20 mg/kg daily for 8 days, had adverse effects on infected goats and camels with *T. evansi* (Trypanosoma evansi) in Sudan. This dosage regimen not only aggravated the condition of the animals but also appeared toxic, ultimately leading to their death. This highlights the critical importance of appropriate dosing, careful monitoring of drug administration, and the potential need for alternative treatment strategies or veterinary oversight to ensure the safety and effectiveness of therapeutic interventions in livestock (43).

#### 3.2.4 Report of adverse drug event: lesson from Tanzania

The Tanzanian Food and Drug Administration (TFDA)^2^ under the Ministry of Health has the mandate to approve and register all medicinal products (biologicals and pharmaceuticals). The TFDA appoints inspectors and orders inspection of premises as well as promotes rational use of drugs and medical devices (43).

Apart from TFDA, the Veterinary Council of Tanzania (VCT) is similar in function, focusing on the registration and licensing of animal health service providers. The VCT also ensures that veterinary professionals meet the required standards of practice. These bodies are essential for maintaining high standards in veterinary medicine and safeguarding animal and public health across these regions. Each has its own approach, but they share the common goal of ensuring that veterinary products and services are effective and safe (44). The agency also works on applications for renewal of registration on reports of additional adverse drug reactions, if any.

#### 3.2.5 Pharmacovigilance and adverse veterinary drug events: lesson from Uganda

The country has a significant number of registered veterinary medicines (421) and a wide distribution network with 1,125 licensed veterinary drug outlets as of 29 May 2020. This level of availability and distribution is crucial for ensuring that quality medicines are accessible for the treatment and prevention of diseases in the animal sector. Through pharmacovigilance activities, veterinary medicines are also actively monitored after use to determine the risk benefit of these products (45). The country reports that the public is increasingly collaborating with the NDA by vigilantly reporting adverse drug reactions occurring in animals, illegal veterinary drug operators, and forwarding quality and safety complaints.

**Figure 1:**
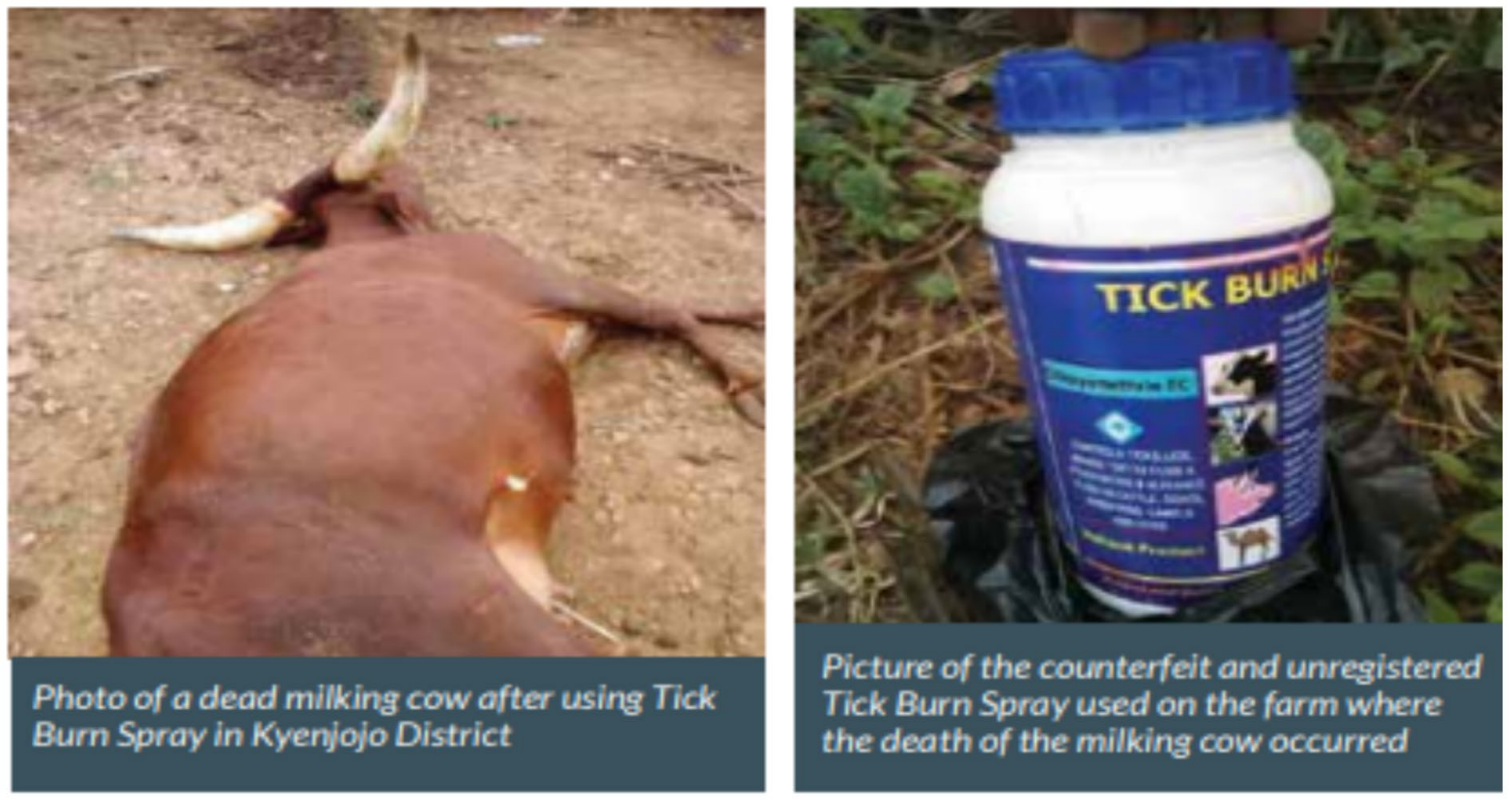
Adverse drug event caused by Tick Burn Spray report from Uganda NDA in 2021.

Uganda’s National Drug Authority (NDA) launching the Med Safety Mobile App for reporting adverse drug reactions (ADRs) in human patients is a significant step forward in pharmacovigilance. Extending this technology to include reporting ADRs in animal patients can serve as a model for other East African countries (45). Regarding reporting practice, the country is doing well in documenting the adverse drug events that are reported to the NDA, which contributes to regulatory decisions and actions for improved safe use of veterinary medicines. An adverse drug event is any observation in animals, whether or not considered product-related, that is unfavorable and unintended and that occurs after any use of a veterinary medicinal product. The National Drug Authority received reports of the circulation of counterfeited and unregistered “Tick Burn Spray,” which caused serious adverse effects to both the animals and the human population exposed to this suspicious product. A particular report from Kyenjojo indicated that a farmer had lost a milking cow following the use of the tick burn spray (46). Figure 1 illustrates a reported adverse drug event from Uganda involving a counterfeit and unregistered tick burn spray, as documented by the National Drug Authority in 2021.

### 3.3 Safety issues of veterinary drugs in East Africa

The literature highlights several critical factors that can compromise the safety of drugs:

The evidence has revealed several crucial elements that could compromise pharmaceutical safety: Medication interactions can have the following effects: (1) reduce efficacy or unintended effects; (2) some medications may interact with foods or beverages, changing their effectiveness or causing adverse reactions; (3) mistakes in prescription, dispensing, or administering medication can harm patients; (4) genetic variations within individuals or between species can affect how medications are metabolized and their efficacy; and (6) low-quality or falsified drugs may contain hazardous materials, lack active ingredients, or have inadequate packaging, posing serious health risks to patients. Strong regulatory frameworks, efficient pharmacovigilance programs, and strict quality control procedures across the pharmaceutical supply chain are needed to counteract these influences and guarantee the efficacy and safety of drugs for the health of humans and animals.

*Drug–drug interactions:* Interactions between different medications can lead to unintended effects or reduced efficacy.

*Food interactions:* Some medications may interact with certain foods or beverages, altering their effectiveness or causing adverse reactions.

*Overdose or under-dose:* Incorrect dosing, whether too much or too little, can lead to serious health consequences.

*Medication and administration errors:* Mistakes in prescribing, dispensing, or administering medications can result in patient harm.

*Genetic or species variability:* Variations in genetic factors among individuals or differences between species can affect how medications are metabolized and their efficacy.

*Counterfeit and substandard medications:* Poor-quality or falsified drugs may lack active ingredients, contain harmful substances, or have inadequate packaging, posing significant risks to patients.

Addressing these factors requires robust regulatory frameworks, effective pharmacovigilance systems, and stringent quality control measures throughout the pharmaceutical supply chain to ensure the safety and efficacy of medications for human and animal health (6). Poor-quality drug is an issue in Africa, but due to limited studies, the magnitude of this problem has not been adequately quantified. It is estimated that about 50% of the veterinary medications marketed in the regions of Asia and Africa are thought to be substandard and falsified (47). These falsified and substandard medicines may lead to drug toxicity, the development of drug resistance, and the deaths of animals (5). Studies revealed that in Sub-Saharan Africa, there is no mechanism in place to monitor veterinary medications, and the use of counterfeit and non-compliant treatments results in adverse effects on animals (12). According to the study mentioned above, veterinary drug safety, especially in the African region, is seriously affected. A weak medicines regulatory capacity, poor access to registered outlets, and the cost of medical products along with complex manufacturing and trading are some of the potential drivers of substandard and falsified medicines for both animals and humans (48). The quality of veterinary medicines can be compromised during the manufacturing process, distribution, and storage conditions (5). Therefore, safeguarding animal health as well as humans can only be possible through monitoring the safety and quality of veterinary medicinal products (47).

### 3.4 Challenges of veterinary pharmacovigilance: lesson from East Africa

Veterinary pharmacovigilance involves monitoring the safety and efficacy of veterinary medicines post-marketing. It is essential for detecting, assessing, and preventing adverse drug reactions (ADRs) and ensuring the safe use of veterinary products. The challenges and lessons from East Africa compared to other African countries reveal critical insights into improving veterinary pharmacovigilance systems. Establishing a national PV system requires a strong legal framework and political commitment (49, 50). Veterinary pharmacovigilance in East Africa faces significant challenges compared to other African countries.

*Lack of Comprehensive Systems:* East African countries generally lack well-established and documented veterinary pharmacovigilance (PV) systems. There is no comprehensive, well-written documentation or guidelines for veterinary pharmacovigilance.

*Lack of Data:* There is a notable absence of data on veterinary drug safety monitoring. This gap highlights the need for more robust monitoring systems to track adverse events and ensure drug safety in veterinary medicine. Given the absence of veterinary drug safety monitoring data in the area and the frequent use of many medications in both human and veterinary medicines, an overview of the pharmacovigilance system of many studies focuses on pharmaceuticals intended for human use and the gaps in animal medicine (51).

The figures derived from the available literature underscore notable deficiencies and obstacles in the pharmacovigilance frameworks in Sub-Saharan Africa, specifically with regard to the East African veterinary medicine landscape. This review also discussed the key points of African veterinary pharmacovigilance:

*Awareness and Understanding:* A report from Senegal showed that 58% of veterinary practitioners were unaware of the concept of pharmacovigilance (12).

*Regulatory Capacities:* According to the WHO report, there are 54 national medicine regulatory authorities in Africa of varying capacities, but most of them are not capable of performing the key functions expected of national medicine regulatory authorities (30). Of the 46 nations in Sub-Saharan Africa that were examined, 87% lacked a working pharmacovigilance system, 59% had no national policy pertaining to medicine safety, 70% had no laws pertaining to adverse event monitoring, 26% had no national PV center, and 61% had no medicine safety advisory committee (52). An assessment of Pharmacovigilance systems in 26 Sub-Saharan African countries showed that only 8 (30%) countries collected reports on adverse events (30). These figures show how inadequate these nations are at monitoring the safety of pharmaceuticals (53).

In Sudan, pharmacovigilance and medication safety have a long history and have gone through numerous stages of development. In 2007, the Federal Pharmacy and Poison Board (FPPB) became the National Medicines and Poisons Board (NMPB), which is currently the executive regulatory body responsible for the legislation, guidelines, and quality of human and veterinary drugs, other aspects involved in the regulation of medications, and the regulation of medical devices (54). From the standpoint of drug safety, the goal of the NMPB is to guarantee the efficacy and safety of pharmaceuticals. Sudan joined the WHO-UMC as a full member in 2008 (54). Sudan, being a full member of the WHO program, utilizes the spontaneous reporting tool available through the Uppsala Monitoring Centre (UMC) website for reporting adverse drug reactions (47). This highlights the importance of having clear and published national policies or guidelines for both human and veterinary pharmacovigilance (PV) across all countries. In East Africa, there is an initiative for the development of pharmacovigilance systems. However, there were no recorded data, particularly related to monitoring the safety issues of veterinary medicinal products. It is important to continually monitor a veterinary medicinal product for safety, quality, and efficacy after it moves from development and registration/approval into the wider population. The information collected through pharmacovigilance systems allows the ongoing assessment of the benefits and risks of the veterinary medicinal product in relation to its target population and throughout its life cycle. The existence of a reliable pharmacovigilance system supports the benefit/risk assessment approach to licensing ‘safe and effective’ medicines through the analytical review of data supporting the quality, safety, and efficacy of the products (33).

### 3.5 Application of pharmacovigilance in the veterinary health sector

The principles and objectives of pharmacovigilance for human medications can be effectively adapted to veterinary settings (27). The role of veterinary pharmacovigilance are critical in livestock landscape such as (1) Detect and quantify previously unknown adverse drug reactions (ADRs) in animals; (2)Determine which groups of animals are most susceptible to negative medication responses based on species, breed, age, gender, physiological status, and underlying health condition; (3) Maintain ongoing assessment of a product’s safety across all approved species to ensure an appropriate balance between risks and benefits. This includes expanding monitoring to additional species and indication; (4) Compare ADR profiles within and between species for medications within the same therapeutic class; (5) Identify and address instances of inappropriate prescribing and dispensing of veterinary medications; (6) Conduct further studies on the toxicological, pharmacological, or microbiological properties of a drug to understand the mechanisms behind adverse reactions; (7) Identify interactions between drugs, especially when new drugs are administered alongside established products or other new drugs; (8) Recognize early signs of unknown safety issues, unexpected therapeutic benefits, and increases in known adverse effects. This entails determining risk factors, estimating risks, and reducing unnecessary pain and suffering of animals (55).

When an adverse effect from a medication is identified, it is crucial to promptly collect and analyze the information. This evaluation aims to reduce future risks associated with the product, ensuring safer usage in veterinary practice. These objectives emphasize the importance of a well-organized pharmacovigilance system in the veterinary sector to improve the overall safety and efficacy of veterinary drugs, thereby protecting both animal and human health as well as the environment (27).

### 3.6 Prospects for future improvements

The assessment aimed to evaluate whether the East African nations were making significant progress in the development of veterinary pharmacovigilance guidelines and addressing veterinary drug adverse events, but certain aspects were left unattended. The effectiveness of the system was hindered by the lack of both active and passive surveillance data, insufficient awareness and knowledge in veterinary healthcare, as well as the limited engagement of regulatory bodies, stakeholders, and academia, thus impeding its advancement (12). Enhancing veterinary pharmacovigilance in East African countries requires a multifaceted approach. Below are potential roadmaps for future enhancements:

#### 3.6.1 Development and implementation of guidelines

Drafting and distributing comprehensive guidelines that address both passive and active surveillance methods for veterinary pharmacovigilance. Furthermore, making certain that the recommendations are updated on a regular basis to take into account the most recent advancements in science and regulation.

#### 3.6.2 Strengthening data collection systems

Putting in place reliable active surveillance mechanisms is vital for quickly identifying and documenting negative occurrences. Enhancing passive surveillance methods further by promoting and streamlining the reporting procedure for farmers and veterinarians. Moreover, creating a centralized database that is available to all stakeholders for the collection and analysis of adverse event reports.

#### 3.6.3 Raising awareness and education

Conducting awareness campaigns to educate veterinarians, farmers, and other stakeholders about the importance of pharmacovigilance and how to report adverse events. Apart from this, offering regular training programs for veterinary professionals on recognizing, managing, and reporting adverse drug reactions. Furthermore, engaging with all stakeholders, including regulatory bodies, veterinary associations, and academic institutions are critical to promote a culture of safety and vigilance.

#### 3.6.4 Enhancing regulatory involvement

Supplying tools and assistance to improve regulatory agencies’ ability to oversee and implement pharmacovigilance procedures. Moreover, encouraging cooperation between academic institutions, industry players, and regulatory bodies to guarantee a unified strategy for veterinary pharmacovigilance. Additionally, treating guidelines that require the reporting of unfavorable incidents and guarantee compliance with frequent audits and inspections is another critical roadmap for veterinary pharmacovigilance.

#### 3.6.5 Leveraging technology

Implementing digital reporting systems to streamline the process of reporting adverse events and ensure timely data collection. Additionally, utilizing data analytics and artificial intelligence to identify patterns and trends in adverse drug event reports, enabling proactive measures to be taken. Indeed, developing mobile applications for veterinarians and farmers are critical to easily report adverse events and access pharmacovigilance resources.

#### 3.6.6 Academic and research collaboration

Encouraging partnerships between regulatory bodies and academic institutions to conduct research on veterinary pharmacovigilance and adverse drug reactions. Additionally, support capacity building initiatives in academic institutions to train the next generation of veterinary pharmacovigilance experts. Furthermore, Promoting the publication and dissemination of research findings to inform policy and practice.

#### 3.6.7 International collaboration

Aligning national pharmacovigilance practices with international standards and guidelines to ensure consistency and improve the quality of data collected. Additionally, Participating in international forums and knowledge-sharing platforms to learn from best practices and experiences of other countries.

## 4 Limitation of the review

The review provides valuable insights into the veterinary pharmacovigilance landscape in East Africa, but it also has several limitations. The review primarily relies on published and grey literature, which may not capture all the relevant data, especially in regions with limited documentation and reporting practices.

The absence of comprehensive and systematically collected data may affect the generalizability of the findings. The focus on selected East African countries means that the findings may not be applicable to other regions, even within Africa. The variability in regulatory frameworks, economic conditions, and healthcare infrastructure across different countries can result in significant differences in pharmaceutical vigilance practices. Since this was the first review of its kind, there isn’t much historical information to go by. This restriction makes it challenging to evaluate long-term trends or track advancements in pharmaceutical practice improvement. Because existing literature was used, there can be variations in the consistency and quality of the sources. Certain sources can have biases or methodological flaws that have an impact on the review’s overall conclusions.

The review emphasizes that major obstacles to successful pharmacovigilance include limited infrastructure, political unpredictability, and inadequate education systems. Because of these problems’ complexity and diversity, it is challenging to suggest simple fixes based on the information at hand.

## 5 Conclusion

The review sheds light on the fact that spontaneous reporting of suspected adverse veterinary drug reactions (ADRs) is the cornerstone of veterinary pharmacovigilance. In East African nations, there was a notable lack of documented information on monitoring the safety of veterinary drugs. Overall, the implementation of veterinary pharmacovigilance and adverse drug event reporting systems were insufficient, necessitating further research and enhanced regulatory collaboration for progress. The review also investigated how many adverse effects of veterinary drugs remain unidentified or are considered insignificant due to underreporting and a general lack of awareness. To address these challenges, collaboration among policymakers, law enforcement, customs officials, regulatory bodies, scientists, pharmaceutical companies, veterinarians, and the public is crucial. Such collective efforts are necessary to develop and enforce veterinary pharmacovigilance guidelines at local, national, and international levels.

## Footnotes

^1^ www.nmpb.gov.sd

^2^ www.tfda.or.tz
